# A novel, dynamic pattern-based analysis of NF-κB binding during the priming phase of liver regeneration reveals switch-like functional regulation of target genes

**DOI:** 10.3389/fphys.2015.00189

**Published:** 2015-07-07

**Authors:** Daniel J. Cook, Biswanath Patra, Lakshmi Kuttippurathu, Jan B. Hoek, Rajanikanth Vadigepalli

**Affiliations:** ^1^Department of Pathology, Anatomy and Cell Biology, Daniel Baugh Institute for Functional Genomics/Computational Biology, Thomas Jefferson UniversityPhiladelphia, PA, USA; ^2^Department of Chemical and Biomolecular Engineering, University of DelawareNewark, DE, USA

**Keywords:** NF-κB, liver regeneration, ChIP-chip, transcription factor binding, priming

## Abstract

Following partial hepatectomy, a coordinated series of molecular events occurs to regulate hepatocyte entry into the cell cycle to recover lost mass. In rats during the first 6 h following resection, hepatocytes are primed by a tightly controlled cytokine response to prepare hepatocytes to begin replication. Although it appears to be a critical element driving regeneration, the cytokine response to resection has not yet been fully characterized. Specifically, the role of one of the key response elements to cytokine signaling (NF-κB) remains incompletely characterized. In this study, we present a novel, genome-wide, pattern-based analysis characterizing NF-κB binding during the priming phase of liver regeneration. We interrogated the dynamic regulation of priming by NF-κB through categorizing NF-κB binding in different temporal profiles: immediate sustained response, early transient response, and delayed response to partial hepatectomy. We then identified functional regulation of NF-κB binding by relating the temporal response profile to differential gene expression. We found that NF-κB bound genes govern negative regulation of cell growth and inflammatory response immediately following hepatectomy. NF-κB also transiently regulates genes responsible for lipid biosynthesis and transport as well as induction of apoptosis following hepatectomy. By the end of the priming phase, NF-κB regulation of genes involved in inflammatory response, negative regulation of cell death, and extracellular structure organization became prominent. These results suggest that NF-κB regulates target genes through binding and unbinding in immediate, transient, and delayed patterns. Such dynamic switch-like patterns of NF-κB binding may govern different functional transitions that drive the onset of regeneration.

## Introduction

Liver resection followed by regeneration is used in the clinic for a variety of conditions, including treatment of hepatocellular carcinoma and live liver transplant. Despite its clinical relevance and decades of study, however, the molecular mechanisms governing liver regeneration following resection remain incompletely characterized. In the laboratory, 70% partial hepatectomy (PHx) of rodents has become a standard model to study liver resection and regulation. PHx provides an ideal model to study the liver's regenerative response because the regenerative stimulus is precisely defined and cell proliferation can be studied without the influence of parenchymal injury or excessive inflammatory cell infiltration.

Liver regeneration following partial resection typically follows a well-documented set of steps involving hepatocytes and non-parenchymal cells (Michalopoulos, 1990; Michalopoulos and DeFrances, 1997; Diaz-Munoz et al., 1998; Monga et al., 2001; Taub, 2004; Grumm et al., 2008). Following PHx, the liver initiates a recovery program inducing hepatocytes to enter the cell cycle and recover lost mass. This recovery program can be thought of as proceeding in four phases: the priming phase (approximately 0–6 h), the hepatocyte replication phase (approximately 12–72 h), the non-parenchymal cell replication phase (approximately 48–96 h), and the termination phase (approximately 96–168 h post-PHx). As early as 30 s after injury, hepatocytes sense the injury due to some combination of increased portal pressure, increased blood flow and shear rate, and increased metabolic demand per hepatocyte or increased toxin load per hepatocyte. These immediate changes cause hepatocytes to release ATP, initiate calcium signaling, and begin WNT signaling. During the remainder of the priming phase, a pro-inflammatory response mediated predominantly through the Kupffer cells primes hepatocytes to enter the cell cycle as a response to the immediate PHx response. Following the priming phase, the hepatocyte replicating phase begins when hepatocytes enter the cell cycle. This phase is characterized by matrix remodeling, high levels of growth factors, and an increase in hepatocyte number. Once hepatocytes have replaced much of the lost liver mass, non-parenchymal cells begin to regenerate. This non-parenchymal cell regeneration phase is characterized by increased levels of VEGF, non-parenchymal cell replication, and increased angiogenesis. As recovery of lost liver mass completes, the termination phase begins. This phase is less well understood, but is thought to occur through a combination of physical size constraints, metabolic constraints, hepatic signaling, and an accumulation of extracellular matrix (Michalopoulos, 2010).

Early in the priming phase, the liver initiates an activation of the innate immune response, which is predominantly driven by cytokines derived from Kupffer cells but may be complimented by production of cytokines and other mediators by sinusoidal endothelial cells, hepatic stellate cells, parenchymal cells, and other resident cell types (Taub, 2004; Michalopoulos, 2007). Among the early intrahepatic intercellular signals detectable following PHx is an increase in pro-inflammatory cytokines, including TNF-α (Michalopoulos, 2007). This early cytokine signaling induces a response in hepatocytes (and other hepatic cells) that includes NF-κB signaling and downstream transcription of NF-κB target genes (Juskeviciute et al., 2008). These NF-κB target genes have been implicated in priming hepatocytes to enter the cell cycle (Michalopoulos, 2007). Previous studies of liver regeneration in rats have shown that an increase in NF-κB binding activity was detectable in whole-tissue extracts as early as 30 min following carbon tetrachloride (CCl_4_) injection, peaked at approximately 1 h post injection, and gradually decreased activity until beyond 48 h post injection, suggesting that NF-κB acts predominantly during the priming phase of liver regeneration (Salazar-Montes et al., 2006). Additionally, after 70% PHx in rats, NF-κB activation was evident within 15 min of surgery, peaked at 1 h post-PHx, remained elevated at 2 h post-PHx, and decreased to near baseline levels at 4 h post-PHx before increasing again at 6 h post-PHx, suggesting that NF-κB may play several roles during early priming, late priming, and early G1 phase of the cell cycle, possibly representing activation in different cell types in the liver (Juskeviciute et al., 2008).

When NF-κB signaling is disrupted, the regenerative ability of the liver is impaired. Although genetically deleting the p50 subunit of NF-κB caused no deficiencies in liver regeneration following CCl_4_ injection, there was a compensation of overall NF-κB activity by increased levels of the p65 subunit (DeAngelis et al., 2001). We can speculate that following deletion of the p50 subunit, the p65 subunit may bind the same genes normally bound by the p50 subunit. Similarly, inactivation of the p65 subunit through a conditional knockout also did not impair liver regeneration (Ringelhan et al., 2012). In this case, NF-κB p50 may have compensated for the depleted p65 subunit. These results suggest that, although the NF-κB p65/p50 dimer is known to regulate immune response, there may be a compensatory effect if only one of the NF-κB p50 and p65 subunits is available during liver repair (Hayden and Ghosh, 2004). As these studies suggest, when all NF-κB signaling was suppressed by inducing IkBa (an inhibitor of NF-κB), hepatocyte proliferation following PHx was decreased resulting in impaired liver regeneration (Yang et al., 2005). The authors of this study suggested that the impaired regeneration was mediated primarily by decreased Kupffer cell activation and IL-6 signaling. These results underscore the importance of understanding the binding of each NF-κB isoform. Our present study focuses on identifying the dynamic binding targets of NF-κB p65 during liver regeneration.

Despite its important role governing liver regeneration, the targets of NF-κB during liver regeneration have not been adequately characterized. In this study we report a genome-wide analysis of NF-κB binding during the priming phase of liver regeneration. We used chromatin immunoprecipitation followed by microarray analysis (ChIP-chip) to identify how NF-κB dynamically binds to genes following PHx in rats. We then related this binding to previously published profiles of gene expression in hepatectomized rats to identify how the dynamic profiles of NF-κB binding relates to regulation of gene expression. Our study demonstrates that NF-κB binding can be classified into dynamic binding switches. Furthermore, these switches appear to dynamically regulate functional pathways associated with liver regeneration. Therefore, NF-κB activation should be interpreted in the context of its dynamic functional binding during liver regeneration.

## Methods

### Animals

All animal studies were approved by the Institutional Animal Care and Use Committee (IACUC) at Thomas Jefferson University. Jefferson's IACUC is accredited by the Association for Assessment and Accreditation of Laboratory Animal Care and experiments were designed using the Guide for the Care and Use of Laboratory Animals.

Adult (8–10 week old) Sprague-Dawley rats were given ad-libitum access to food (Chow) and water. When their weight reached 275–350 g, they were anesthetized and subjected to 70% PHx by surgical removal of medial and left lateral lobes as per standard procedure (Higgins and Anderson, 1931; Juskeviciute et al., 2008). The medial and left lateral lobes were flash frozen using liquid nitrogen-cooled aluminum clamps to serve as within-animal, 0 h controls. At 1, 2, 4, and 6 h post-PHx, rats were again anesthetized and the remnant liver tissue was excised and flash-frozen as before. Following excision of the remaining liver mass, rats were sacrificed by cervical dislocation. Liver tissue from 0, 1, 2, 4, and 6 h post-PHx was subjected to chromatin immunoprecipitation. Immunoprecipitated samples (ChIP) from 0, 1, and 6 h post-PHx were used to identify genome-wide NF-κB binding sites using microarrays (ChIP-chip). Immunoprecipitated samples from 0, 1, 2, 4, and 6 h post-PHx were used for validation and extension of ChIP-chip results using qPCR.

### Chromatin immunoprecipitation (ChIP)

Chromatin immunoprecipitation (ChIP) assays were performed using total liver tissue to map the *in vivo* distribution of NF-κB/DNA interactions using a Magna ChIP G Chromatin Immunoprecipitation kit (Merck Millipore) according to manufacturer's instructions. Approximately 50 μg minced liver tissue was fixed for 10 min with 1% formaldehyde, which crosslinks DNA and chromatin binding proteins to ensure co-immunoprecipitation. Glycine (1× in accordance with the EMD Millipore protocol) was then added to quench unreacted formaldehyde. Cells were lysed and chromatin was sheared by sonication to generate fragments of 200–1000 bp (40 min. of sonication using a 30 s on, 30 s off cycle). Fragments bound by NF-κB were immunoprecipitated using a ChIP-grade NF-κB antibody (Cat#ab7970, Rabbit polyclonal NF-κB p65 antibody and negative control IgG antibody from Abcam Inc., Cambridge, MA) in combination with Protein G conjugated solid support matrix magnetic beads enriched for the antibody of interest using electrophoresis on a 1% agarose gel. This NF-κB antibody has been employed extensively in previous studies to specifically detect NF-κB proteins in Western blots and immunohistochemistry assays, as well as for chromatin immunoprecipitation assays (Burdelya et al., 2013; Kasama et al., 2014; Luo et al., 2014). Negative controls in the absence of the primary antibody showed negligible signal intensity (Figure S8).

### Identification of NF-κB binding sites using microarrays (ChIP-chip)

Purified ChIP DNA was amplified using the GenomePlex Complete Whole Genome Amplification (WGA) kit from Sigma that allows for nearly 500-fold amplification of genomic DNA using OmniPlex Library molecules flanked by universal priming sites. Genome-wide promoter enrichment was measured using the Roche Nimblegen Promoter array platform, which has 720,000 probes (Rat ChIP-chip 3x720K RefSeq Promoter Arrays—3 identical arrays per glass slide with 72,000 probes per array, Roche NimbleGen, Inc., 504 South Rosa Road, Madison, WI). Experimental ChIP and total DNA samples are labeled using 9-mer primers that have Cy3 and Cy5 dyes attached and Klenow added. The labeled experimental ChIP and total DNA samples were co-hybridized to the array for 16–20 h at 420°C, washed, and scanned using an Agilent scanner (Agilent Technologies) following manufacturer instructions.

Array images were used for data extraction as paired files; genomic feature format files were then produced for analysis of scaled log2-ratio data. The intensity ratio of immunopreciptated to total DNA (not taken through immunoprecipitation steps) was calculated at each genomic position to identify regions where increased signal (i.e., DNA fragment enrichment) was observed relative to the control sample. Peak regions identified as statistically significant binding sites were generated from the scaled log_2_-ratio data, and peaks were mapped to the nearest gene's transcription start site.

Roche NimbleGen arrays were used because their proprietary, light-mediated synthesis process produces high-density microarrays of long oligonucleotide probes (50–75 mer). These long oligo arrays, when used in combination with high-stringency hybridization protocols, produce results of unparalleled sensitivity and specificity. In addition, because Roche NimbleGen performs ChIP-chip experiments using a two-color protocol, where control and test samples are co-hybridized to the same array, inter-array variation is eliminated. As a result, NimbleGen ChIP-chip service can readily detect enrichment as low as 2-fold of the target binding site in a ChIP sample.

Data generated from these experiments as well as processed data used to draw the conclusions of this study were deposited in the Gene Expression Omnibus repository and are publically available at www.geo.ncbi.org.

### Genome-wide mapping and peak detection: Identification of binding sites

The possible binding regimes (peaks) that correspond to the binding targets were detected if 4 or more probes showed a signal above the cutoff value ranging from 90 to 15% using a 500 bp sliding window. Cut-off values were set as a fraction of the hypothetical maximum signal (mean signal plus six standard deviations). An empirical false discovery rate (FDR) was calculated for each peak using a bootstrapping method, which randomized the probe signals 20 times. The calculated FDR is an approximation of the probability of a false positive. To minimize false positives while maximizing NF-κB binding signal, an FDR cutoff of 0.05 was used for all analyses (See Figure S1). Any FDR cutoff higher than 0.05 would have led to an exponentially increased number of false positive NF-κB bound genes. Although steps were taken to limit false positives, some of these binding sites identified during peak detection might mediate higher-order genomic interactions and influence chromosome structural modifications.

### Peak annotation

Peaks were annotated with candidate target genes with the assumption that the distance between a center of binding peak and the transcription start site (TSS) of the gene is shorter than a threshold cutoff. We defined these “gene regions” as spanning from 5 kb upstream of the TSS to 1.5 kb downstream of the end of transcription. The peak files were annotated with Ensembl version 5.0 (Rnor_5.0) transcript genes using a 5000 base pair cutoff distance from the TSS using the Chip Peak Anno Bioconductor package in R (Gentleman et al., 2004; Zhu et al., 2010; R Core Team, 2013). The peaks were then annotated with detailed characteristic genomic features: peak region location, gene annotation of nearest transcript (intron, exon, intragenic, etc.), chromosome, start and end of genes, nearest transcripts and transcript boundaries, distance from TSS, RefSeq IDs, Entrez IDs, TSS, trophoblast-specific element (TSE), and average phastcon scores obtained.

### Dynamic pattern analysis

We used a dynamic pattern-based strategy to analyze the dynamic NF-κB binding post-PHx. We first discretized NF-κB binding by identifying genes as bound (1) or unbound (0) at each time point post-PHx. To be identified as bound, NF-κB binding had to be seen in 2 out of 3 biological replicates at a time point (FDR ≤ 0.05). Additionally, we analyzed how our choice of FDR cutoff would influence our pattern-based analysis and found that choice of FDR cutoff has minimal effect on the fraction of NF-κB bound genes in each of several binding pattern (Figure S2). We found that setting the FDR cutoff too low appeared to remove useful pathways from the analysis, but setting the cutoff to high appeared to include pathways that may represent data from false positives (Tables S1–S3). An FDR cutoff of 0.05 struck a balance between these extremes. Because discretized NF-κB binding has 2 distinct states (0, unbound; 1, bound) and we analyzed 3 time points, we generated 8 (2^3^) dynamic binding patterns where NF-κB was identified as bound or unbound at each time: 000, 001, 010, 011, 100, 101, 110, 111. Each pattern was then described by both a binary string (i.e., 011) and its digital representation (i.e., Pattern 3). Because Pattern 0 contains no NF-κB bound genes, it was not considered in the subsequent analyses. Each gene bound by NF-κB was sorted into its binding pattern. Because the patterns are mutually exclusive, each binding pattern of each gene can match only one binding pattern.

### Integration with expression data

Because transcription factor binding does not cause immediate changes in gene expression level, we looked for previously published gene expression data from time points after 1 and 6 h post-PHx. Illumina RNA-seq gene expression data for the liver following PHx was downloaded from GEO (Accession: GSE54673) (Edgar et al., 2002). Normalized average gene expression for 0 h (baseline), 4 h (following 1 h NF-κB binding), and 12 h (following 6 h NF-κB binding) post-PHx was reported in reads per million mapped reads (RPKM) (Naugler, 2014). Fold change over baseline was calculated for each time point by subtracting the log-transformed 0 h expression value from the corresponding datum at 4 or 12 h post-PHx. Genes were identified as differentially expressed if they had a fold-change ≥2 above or below baseline (Figure S3). Gene IDs from the expression data were converted to Refseq mRNA IDs using Clone/Gene ID Converter (Alibes et al., 2007). Each gene bound by NF-κB was then matched to a differentially expressed gene from the RNA-seq dataset using the RefSeq ID. Unless otherwise specified, non-differentially expressed genes were excluded from further analyses.

### Pathway analysis and functional association identification

Functional associations between genes in each binding pattern were carried out using the online pathway analysis tool DAVID (Database for Annotation, Visualization and Integrated Discovery) (Huang et al., 2009). Because DAVID v6.7 provides a comprehensive set of functional annotation tools for investigators to understand biological meaning behind large list of genes, the DAVID software was used to identify enriched biological functions for each NF-κB binding pattern, particularly focused on gene ontology (GO) terms and KEGG pathways (Ashburner et al., 2000; Kanehisa and Goto, 2000; Kanehisa et al., 2014). A clustering *p*-value cutoff of 0.05 was used to filter the gene function list to only functions highly enriched in each pattern.

### Motif discovery

To find the binding sites enriched by NF-κB along with other transcription factors potentially associated with transcriptional regulation during liver regeneration, we used the *de novo* motif discovery program DME (Discriminating Motif Enumerator) using default parameters and a string length of 10 (Smith et al., 2005). DME is particularly well suited to our analysis because it is a *de novo* motif discovery program based on an enumerative algorithm that identifies optimal motifs from a discrete space of matrices with a specific lower bound on information content. It is therefore well suited for analyzing large datasets. This string length was chosen as 10 because it robustly identified NF-κB as a major binding motif in our data.

Peaks were filtered prior to using DME as follows. First, only peaks with an FDR ≤ 0.05 in at least 2 out of 3 biological replicates were considered in the analysis. Next, the peaks with the strongest peak scores were chosen for final analysis. Scree plots of peak score were used to identify appropriate peak score cutoffs for each time point (Figure S4).

Once *de novo* motifs were found, associated transcription factors were predicted by scanning the motifs against the TRANSFAC databases containing documented transcription factor binding sites using the clustering program STAMP with default parameters matching to TRANSFAC v11.3 (Matys et al., 2006; Mahony and Benos, 2007). Any motifs co-occurring with the NF-κB binding motif were considered to be potential co-regulators with NF-κB of a particular set of genes.

### Quantitative PCR validation of NF-κB binding

Quantitative PCR primer sets for 21 genes across all 8 NF-κB binding patterns were designed using TRANSFAC, a Biobase software (Matys et al., 2006). Quantitative analysis of promoter binding was performed through real-time PCR on an ABI Prism 7000 (Applied Biosystems, Foster City, CA) according to manufacturer's instructions and using iTaq SYBR Green super mix from Bio-rad (Bio-Rad Laboratories, Inc., 2000 Alfred Nobel Drive, Herles, CA 94547). Primer sequences can be found in Table S4.

## Results

### NF-κB binding following partial hepatectomy

**Figure 1:**
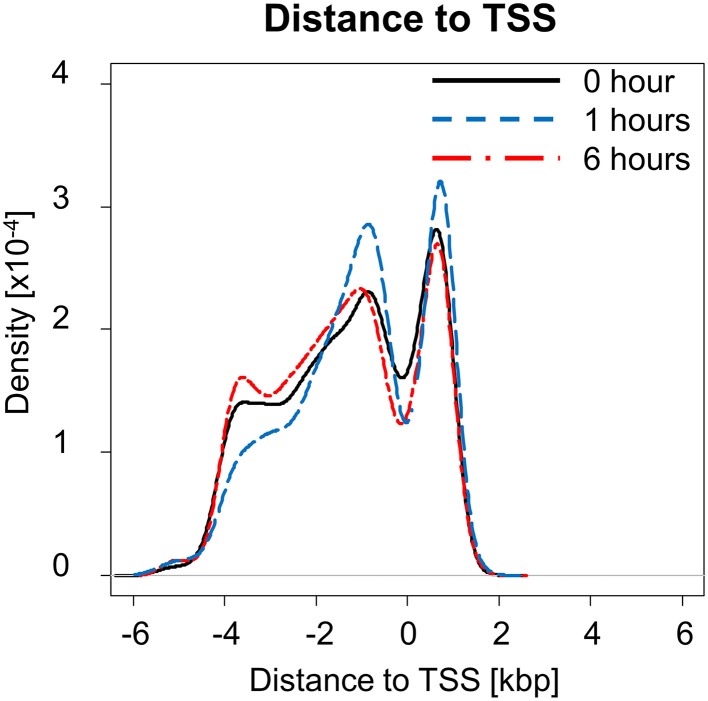
**Distance to transcription start site (TSS) at each time measured post-PHx**. At 1 h post-PHx, the peak distance to TSS appears to decrease with more NF-κB binding close to the regulated gene both upstream and downstream.

NF-κB is an important regulator and mediator of cytokine response to injury. We investigated how NF-κB binding responded to PHx by quantifying the number of genes bound by NF-κB at each time measured post-PHx. The genes bound at each time point included both genes unique to that time point and common among time points. We found that the total number of genes bound by NF-κB during the first 6 h post-PHx was similar at each time point investigated, with 2518 genes bound by NF-κB at baseline (0 h), 2440 genes bound at 1 h post-PHx, and 2396 genes bound at 6 h post-PHx (using an FDR cutoff ≤0.05), although, as the priming phase progressed, the numbers of genes bound by NF-κB decreased. Thus, although NF-κB has been shown to become activated quickly following PHx and maintain its activity until at least the end of the priming phase, the overall number of genes bound by NF-κB during the priming phase of liver regeneration remained similar in our analysis. What changed, however, was the distance from the binding site to the transcription start site (TSS) of the nearest gene (Figure 1). At 0 and 6 h post-PHx, NF-κB binding occurs with two peaks with similar frequency: one upstream of the TSS and one downstream. At 1 h post-PHx, NF-κB binds to both upstream and downstream of the TSS but the downstream binding is clustered closer to the TSS than for 0 and 6 h. Additionally, the upstream and downstream peaks appear higher at 1 h than at 0 or 6 h, indicating that NF-κB binds more genes close to their TSS at 1 h (Figure 1). Transcription factors binding closer to the TSS of a gene have a stronger effect on regulation of gene expression (Cheng and Gerstein, 2012; Cheng et al., 2012). Taken together, our data suggest that at 1 h following PHx NF-κB has a stronger effect on bound genes. This correlates with previous studies showing increased NF-κB activity at 1 h post-PHx (Juskeviciute et al., 2008).

### Dynamic pattern analysis of NF-κB binding

**Figure 2:**
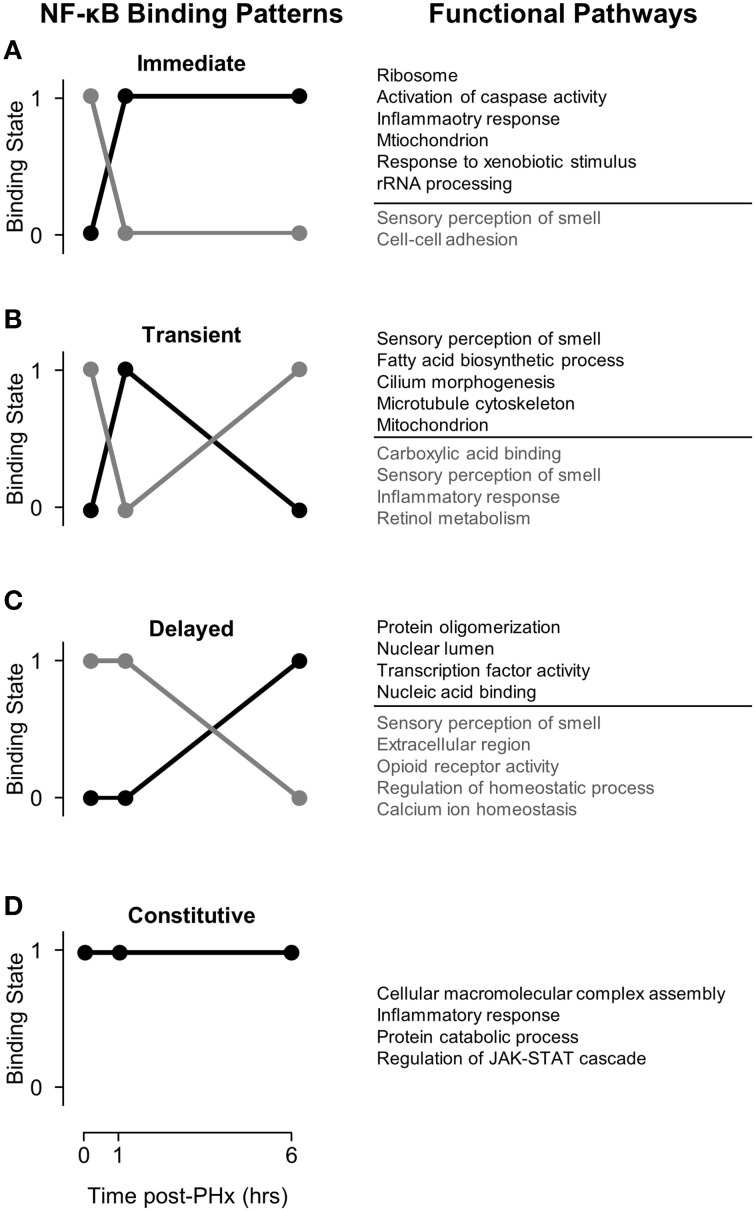
**NF-κB binding patterns with associated pathways found using DAVID. (A)** Immediate binding and unbinding **(B)** Transient Binding and unbinding **(C)** Delayed Binding and Unbinding **(D)** Constitutive Binding. Our analysis revealed that NF-κB binds to many genes involved in sensory perception of smell at baseline. Following PHx, however, these targets become unbound as NF-κB instead targets pathways previously related to be critical to liver regeneration. It is possible that genes involved in sensory perception of smell serve as a sink for NF-κB binding so that transient pulses to NF-κB binding do not start cascades of inflammation.

Activation of pro-inflammatory cytokines during the priming phase is a dynamic process involving multiple regulatory feedbacks. Therefore, we used a dynamic pattern-based strategy to analyze the binding of NF-κB to target genes following PHx. We first discretized NF-κB binding by identifying genes as bound (1) or unbound (0) at each time point post-PHx. To be identified as bound, NF-κB binding had to be seen in 2 out of 3 biological replicates at a time point (FDR ≤ 0.05). Because discretized NF-κB binding has 2 distinct states, we were then able to organize NF-κB binding into 7 dynamic binding patterns (Figure 2). Each pattern is described by both a binary string (i.e., 011) and its digital representation. Therefore, the binding pattern unbound by NF-κB at 0 and 1 h but bound at 6 h post-PHx (binary string 001) becomes pattern 1, the binary string 010 (0 h unbound, 1 h bound, 6 h unbound) becomes pattern 2, and so on until binary string 111 becomes pattern 7 (Table 1).

**Table 1:** **Pattern analysis of NF-κB binding during the priming phase post-PHx**.

**Digital pattern**	**Binary pattern**	**NF-κB bound genes**	**Differentially expressed genes**
1	001	1067	154
2	010	1140	155
3	011	392	53
4	100	1108	155
5	101	502	71
6	110	473	55
7	111	435	190^*^

* *Denotes total number of genes bound by NF-κB & measured in RNA-seq, not differentially expressed genes*.

The number of genes bound by NF-κB in each pattern (Table 1) showed that transient binding (Pattern 2: 010), immediate unbinding (Pattern 4: 100), and delayed binding (Pattern 1: 001) contained the highest number of genes bound by NF-κB. Therefore, the major dynamic effects of PHx on NF-κB during the priming phase appear to be a large unbinding event followed by a transient binding event and a delayed binding event at the end of the priming phase. This suggests that the bound NF-κB in the resting liver is not all bound in an active conformation. It should be noted that these binding sequences may represent a pool of previously available NF-κB that comes unbound from its normal location on the genome and dynamically induces transcription in two distinct sets of genes (one at 1 h post-PHx and one at 6 h post-PHx). All the other binding patterns contained approximately the same number of genes bound by NF-κB (Table 1, Digital patterns 3, 5, 6, and 7). These patterns of NF-κB binding may also considerably regulate early tissue response to PHx. There were also a considerable number of genes constitutively bound by NF-κB (Pattern 7: 111), suggesting that NF-κB binding following PHx continues to maintain functions occurring in non-stressed tissue in addition to dynamically switching binding locations.

### Functional pathways represented by genes bound by NF-κB post-PHx

We first coupled NF-κB dynamic binding patterns into pairs of NF-κB binding switches (Figure 2, left panel). This allowed us to characterize NF-κB binding switches as an immediate response switch (Figure 2A), a transient response switch (Figure 2B), and a delayed response switch (Figure 2C), with constitutive binding also occurring (Figure 2D). We then investigated which functional pathways were bound and unbound by NF-κB during each switch using the DAVID software to identify functional clusters of genes (Huang et al., 2009). One unexpected result was that NF-κB binds to many genes within the functional category sensory perception of smell, specifically prior to PHx and in the “unbinding” (gray) patterns. Following PHx, NF-κB ceased binding to these genes to perform other functions. Overall, we found that NF-κB bound to 510 olfactory receptor genes; of these, 9 were differentially expressed following PHx (at a 2-fold-change cutoff). We investigated if the chromatin was accessible at these genes by comparing our NF-κB binding regions and associated gene region to whole-liver DNAse1 hypersensitivity regions in mouse livers found by the ENCODE project (Yue et al., 2014). We found that the majority of these NF-κB binding sites had no DNAse1 hypersensitivity, with only 8 NF-κB binding sites robustly accessible across animal replicates (a subset of these genes are shown in Figure S5). Of these 8 genes, 6 had inaccessible gene regions (Figure S5A), while 2 had open chromatin (DNAse1 hypersensitivity) at the beginning of the gene region (Figures S5B, C). The accessible genes were not differentially expressed. We speculate that NF-κB binding sites for these sensory perception of smell genes may act as a buffer for NF-κB activation under normal conditions to prevent spurious hepatocyte priming but are made unavailable for NF-κB binding following a sustained challenge such as hepatectomy by mechanisms such as histone methylation or acetylation. Alternatively, recent work has shown functional olfactory receptors in the kidneys of mice which act as chemical sensors and can modulate glomerular filtration rate (Pluznick et al., 2009). It is possible that olfactory receptors in the liver may also act as chemical sensors to modify liver behavior. These results are difficult to interpret at present and require more detailed study.

### NF-κB switches co-ordinate dynamic tissue function post-PHx

**Figure 3:**
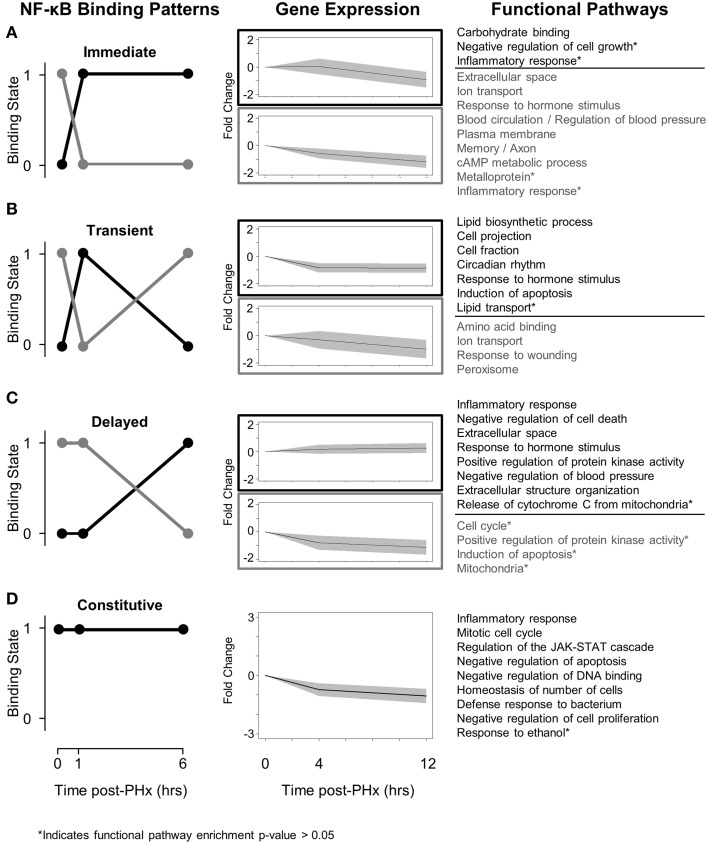
**Switching mechanisms of NF-κB binding post-PHx. (A)** NF-κB immediate response switch. **(B)** NF-κB transient response switch. **(C)** NF-κB delayed switch. **(D)** NF-κB constitutive binding. NF-κB was constitutively bound to genes responsible for cell cycle, cellular response to stress, and the JAK-STAT pathway. The regulation of these genes by NF-κB may be essential for tissue function.

In light of the role of NF-κB in dynamically regulating tissue function post-PHx, we next investigated how NF-κB switches regulated gene expression. We first used the NF-κB switches to organize genes into binding patterns as before (Figure 3, left panel). We then identified gene expression levels of genes bound by NF-κB using a previously published RNA-seq gene expression data set of rat liver regeneration at 0, 4, and 12 h post-PHx (Figure 3, center panel; Table 1). Genes were identified as differentially expressed with a fold-change value ≥2 (see Material and Methods for rationale). Differentially expressed genes corresponding to each NF-κB switch were then analyzed to identify functional clusters of genes using the DAVID software (Figure 3, right panel) (Huang et al., 2009). For constitutive NF-κB binding, all NF-κB bound genes were analyzed rather than only differentially expressed genes. This association of NF-κB binding with subsequent gene expression was somewhat tenuous because of the low sampling frequency and non-identical time points used between studies; however, this approach focused our analysis to NF-κB binding that appears to have a dynamic functional role in gene expression.

We found that although NF-κB binding follows switch-like behavior, gene expression patterns do not all follow the same switching behavior. In all switches analyzed, however, expression levels of those genes unbound by NF-κB tend to decrease while expression levels of those genes bound by NF-κB tend to increase (or decrease less) (Figure 3, Center panel). Several factors may contribute to a weak correlation between binding and gene expression. One factor is the time delay between measured NF-κB binding and measured gene expression. Although it has been well documented that transcription can have delayed effects on gene expression, 3 and 6 h following binding events at 1 and 6 h post-PHx may be too long for robust correlations between transcription factor binding and gene expression (McAdams and Arkin, 1997; Schmitt et al., 2004). Similarly, once transcribed, different mRNA may have different regulation, degradation rates, and half-lives. Additionally, NF-κB may coordinate with other transcription factors known to be active during liver regeneration to dynamically regulate gene expression.

We found that immediately post-PHx NF-κB stopped regulating genes associated with multiple pathways and instead began to regulate genes associated with only a few pathways, predominantly with the functions (GO Terms) negative regulation of cell growth and inflammatory response (Figure 3A). This result suggests that immediately following PHx there may be a limit to NF-κB bioavailability. The binding seen at 1 h post-PHx occurs before there would be significantly increased production of NF-κB protein. This interpretation is consistent with mathematical models which can capture short-term dynamics of NF-κB signaling without considering additional NF-κB production (Lipniacki et al., 2004). Therefore, the available NF-κB may cease to regulate less essential functions like regulation of blood pressure and begin to regulate transcription of genes required to prime hepatocytes for replication. Additionally, the negative regulation of cell growth may ensure that all available metabolic energy goes toward cell replication rather than cell growth to meet functional demand (Shestopaloff, 2014).

Potentially using this same pool of available NF-κB, NF-κB transiently switched from regulating genes associated with amino acid binding, ion transport, and response to wounding, and began to regulate lipid biosynthetic processes, cell projection, circadian rhythm, and induction of apoptosis (Figure 3B). This suggests that NF-κB transiently shifts from governing normal tissue functions (like amino acid binding and ion transport) to governing an additional response to tissue damage that may also modulate normal tissue functions (increasing binding to genes governing response to outside stimuli, circadian rhythm, and induction of apoptosis). The transient NF-κB switch also regulated functions associated predominantly with hepatic stellate cells. Specifically, NF-κB began to regulate lipid synthesis and transport as well as cell projections, potential components of modulating hepatic stellate cell activation. Because hepatic stellate cells are not thought to begin producing growth factors until approximately 12 h post-PHx, this induced NF-κB binding may act as a pioneering signal stimulating a transcriptional cascade that will ensure that hepatic stellate cell activation occurs at the proper time post-PHx (Michalopoulos, 2007).

After there has been sufficient time to begin producing additional NF-κB protein, NF-κB binding undergoes a delayed response switch (Figure 3C). As opposed to the immediate response switch, the delayed switch turns off relatively few pathways and turns on multiple pathways. The pathways switched on included inflammatory response, positive regulation of protein kinase activity, negative regulation of apoptosis, and extracellular structure organization and suggest that by the end of the priming phase NF-κB begins to drive production of genes required for hepatocyte replication, including protein kinases. Additionally, NF-κB appears to switch its role from pro-apoptotic (transient) to anti-apoptotic (delayed). This switch may be an important regulator of liver failure after resection.

NF-κB also displayed constitutive binding to a select set of genes throughout the priming phase (Figure 3D). These genes were normally bound by NF-κB and remained bound despite PHx, indicating that they may be essential for tissue function. They included genes in pathways canonically associated with NF-κB binding, including inflammatory response, regulation of the JAK-STAT signaling cascade, negative regulation of apoptosis, and the mitotic cell cycle. The constitutive binding to genes in these pathways indicates that these pathways, which are typically associated with a dynamic stress, may also be critical for normal tissue function.

### NF-κB binding motif analysis

**Figure 4:**
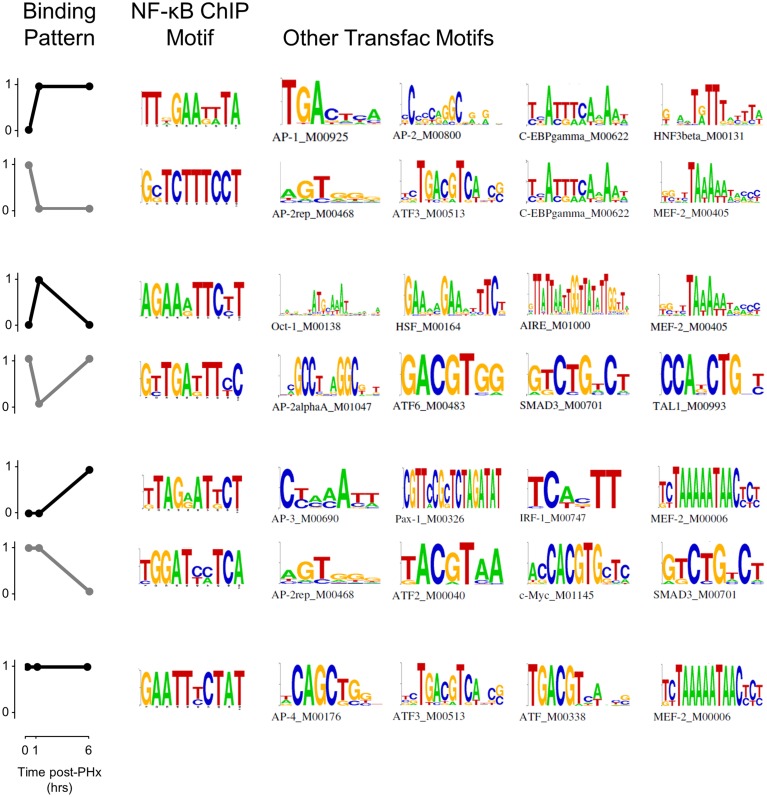
**Pattern-based motif analysis of NF-κB binding sites**. Strongly bound sites in each binding pattern were matched to known transcription factor binding sites using the software DME and the TRANSFAC database. Potential co-regulators of NF-κB binding switches were identified for each binding pattern.

NF-κB binding motif analysis revealed that NF-κB had multiple cofactors that may act to help regulate transcription following PHx (Figure 4, Figure S6). We found several motifs associated with the immediate NF-κB switch in the AP-1 and ATF families. AP-2 in particular may be a cofactor helping to regulate genes within this switch. C/EBP-gamma was also associated with both the binding (black) and unbinding patterns (gray). The binding pattern (black) in the transient response switch was associated with transcription factors related to cellular response to stress (metabolic Oct-1 or heat HSF), immune response (AIRE, HSF) and the cell cycle (MEF-2). Whereas the transient unbinding pattern (gray) was associated with transcription factors related to immediate-early gene expression (AP-2a and ATF6) and those that may relate to transcriptional control of hepatic stellate cell activation (SMAD3 and TAL1). The delayed NF-κB switch was associated with many of these same transcription factors (AP-2, AP-3, ATF, ATF-4, SMAD3, and MEF-2) as well as several additional transcription factors involved in inflammation (IRF-1) and the cell cycle (c-Myc). Constitutive NF-κB binding was associated with similar transcription factors (AP-4, ATF, ATF-3, and MEF-2).

When analyzed at each time point instead of in each pattern, we found multiple transcription factors that may act in concert with NF-κB including IRF-1, IRF-2, IRF-7, ICSBP (IRF-8), HSF, and MEF-2 (Figure S6). The IRF family of transcription factors is predominantly involved in interferon regulation and may be induced as a response to pro-inflammatory signals. IRF-1 is involved in the NF-κB signaling pathway and response to IL-1β. IRF-2 is involved in regulation of transcription and may enhance the function of other IRF transcription factors. IRF-7 is involved in IFNα and IFNβ production. ICSBP (also known as IRF-8) is involved in the granulocyte-macrophage colony stimulating factor signaling pathway and may indicate hepatocyte response to Kupffer cell activation. Similarly, HSF is typically involved in cellular response to heat stress but may also be active in cellular response to other forms of stress or local inflammation/tissue damage. MEF-2 is also involved in the cellular response to calcium changes and may bind as a cofactor to NF-κB to modulate NF-κB activity when calcium signaling increases, as it does following PHx (Diaz-Munoz et al., 1998).

In addition, we found several motifs to be selectively enriched at 6 h post-PHx (Figure S6). These motifs included Cdx-2, HFH4, Pax-4, and PPARa. Cdx-2, HFH4, and Pax-4 are all involved in hepatocyte entry into the cell cycle. Cdx-2 is involved in positive regulation of cell proliferation, HFH4 is involved in cellular response to growth factor stimulus, and Pax-4 is involved in negative regulation of apoptosis. The selective enrichment of these cofactors at 6 h post-PHx correlates with our results indicating that NF-κB may play a role preparing hepatocytes for entry into the cell cycle at the end of the priming phase. In contrast, PPARα binding as a cofactor to NF-κB may indicate that these factors work together to regulate correct timing of hepatic stellate cell activation post-PHx. PPARα is known to regulate fatty acid metabolism (which is increased during stellate cell activation) and may be a therapeutic target to ameliorate alcoholic liver disease, which may proceed through activation of hepatic stellate cells (Friedman, 2008; Nan et al., 2014).

### AP-1 as a co-regulator of NF-κB bound genes following PHx

**Figure 5:**
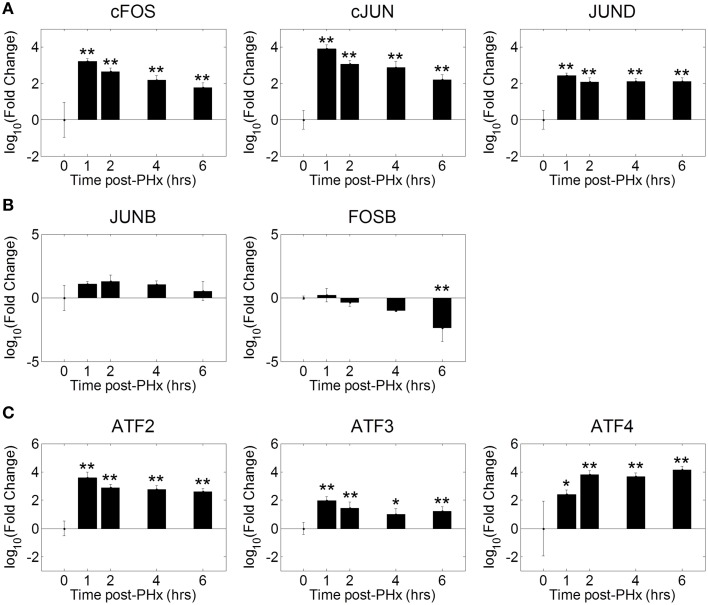
**Co-ordinated activation of AP-1 and ATF family transcription factors during liver regeneration**. Our motif analysis suggested that AP-1 binding coincided with NF-κB binding to co-regulate expression of genes during the priming phase of liver regeneration. The AP-1 family of transcription factors was found to be strongly activated by 1 h post-PHx in rats. This activation was maintained throughout the priming phase for all transcription factors except FOSB and JUNB. FOSB activity decreased following PHx, which JUNB activity transiently increased before returning to baseline levels. ^*^p-value < 0.05, ^**^p-value < 0.01.

Our motif analysis found several motifs enriched in NF-κB binding sites belonging to the AP-1 and ATF transcription factor families. We therefore investigated the dynamics of AP-1 and ATF transcription factor family activities during the priming phase post-PHx. We found that the AP-1 family of transcription factors was strongly activated by 1 h post-PHx in rats and remained activated over the course of the priming phase (Figure 5A). This behavior was true for AP-1 family all transcription factors measured except FOSB and JUNB. FOSB activity decreased following PHx, while JUNB activity showed little change (Figure 5B). ATF family transcription factor activities were also increased above baseline throughout the priming phase (Figure 5C). These results coupled with the potential co-regulatory binding sites identified with NF-κB indicate that, as expected, AP-1 and ATF family transcription factors coordinate with NF-κB in regulating the priming for liver regeneration.

### Validation of selected NF-κB binding dynamics using ChIP-qPCR

**Figure 6:**
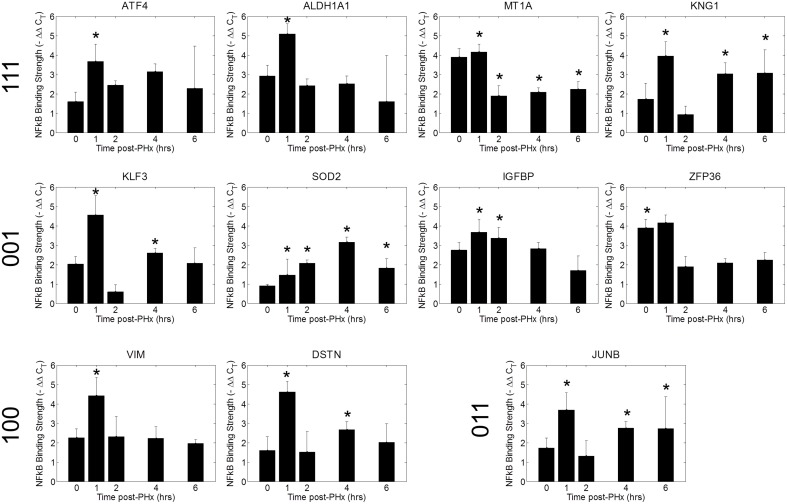
**ChIP qPCR validation**. Binding patterns for selected genes were investigated using ChIP-qPCR. ^*^p-value < 0.05.

We tested a set of 21 of genes through the use of NF-κB ChIP followed by high-throughput qPCR (Figure 6, Figure S7) (Spurgeon et al., 2008). We found that NF-κB bound to the promoter regions of several of these genes with similar dynamics as in the ChIP-chip analysis (Figure 6). The similar binding dynamics seen between platforms supports the results of our ChIP-chip analysis. We further validated our results for promoter enrichment at the Sod2, iNOS, G0S2, and IGFBP1 gene promoter regions using PCR followed by visualization in a 1% agarose gel (Figure S8).

## Discussion

**Figure 7:**
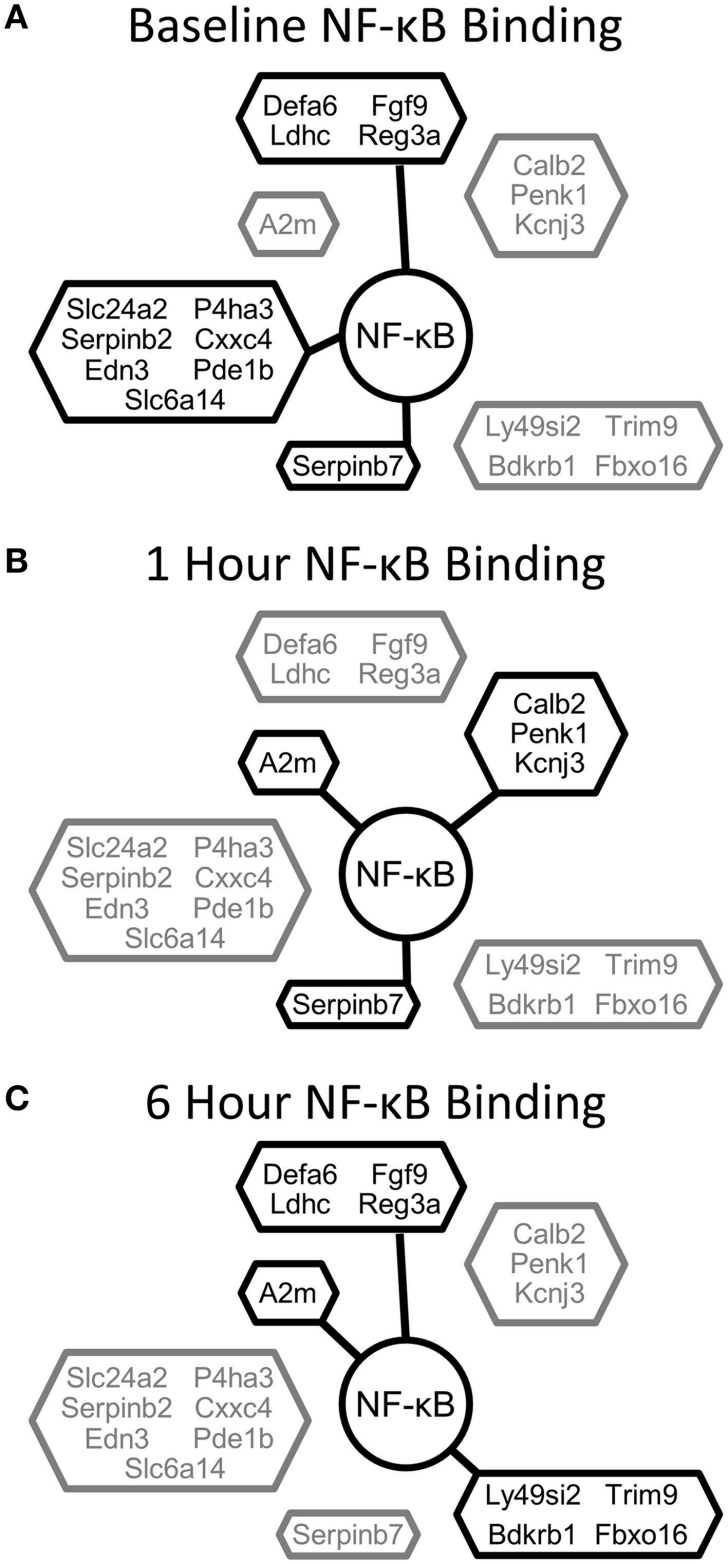
**NF-κB binding for the top 20 differentially expressed genes clustered according to NF-κB binding pattern. (A)** NF-κB binding at baseline, **(B)** NF-κB binding at 1 h post-PHx, **(C)** NF-κB binding at 6 h post-PHx. Black, Bound; Gray, Unbound.

Our results indicate that the acute challenge of a partial hepatectomy causes NF-κB binding in the liver to operate as a dynamic switch regulating tissue function. The immediate, transient, and delayed NF-κB signaling profiles appear to serve different purposes in driving regeneration. Early post-PHx, NF-κB binding transitioned from governing many functions to governing mainly those functions necessary to set hepatocytes up for entry into the cell cycle. These functions were maintained throughout the priming phase. Transiently post-PHx, NF-κB binding transitioned away from binding genes involved in maintaining tissue function and toward binding genes involved in apoptosis, circadian rhythm, and hepatic stellate cell activation. The transient switch may therefore be involved in synchronizing healthy cells for entry into the cell cycle while inducing damaged cells to commit apoptosis. The transient switch may also ensure proper timing of hepatic stellate cell activation following PHx. As the priming phase ended, NF-κB began to regulate many genes setting up hepatocytes to enter the cell cycle. This switch may indicate a role for NF-κB contributing to hepatocyte entry into the cell cycle as regeneration progresses past the priming phase. Additionally, several genes involving mitochondrial function that are bound and unbound in this switch (including Atp5d, NDUFA10, ACSL5, and mfn1) may be important to govern the fraction of metabolic demand delegated to regeneration and that delegated to maintenance of tissue function as hepatocytes enter the cell cycle (Shestopaloff, 2014). The binding patterns of differentially expressed genes with the highest expression (top 20 genes) show dynamic regulation by NF-κB throughout the priming phase (Figure 7).

In addition to the NF-κB binding switches, we observed constitutive binding, where NF-κB was bound before PHx and remained bound throughout the priming phase. Because liver tissue function is maintained during regeneration, it is likely that these genes consist of an essential set of genes required for NF-κB contributions to normal liver function. The pathways constitutively governed by NF-κB suggest that NF-κB could be important for maintaining the balance of the innate immune system in the liver. The innate immune system must be balanced such that it can respond to pathogens or inflammation but not so active that chronic inflammation results. Another possibility is that, because the drivers for priming rely on processes in non-parenchymal cells (especially Kupffer cells), dynamics of NF-κB binding in the innate immune system may not be detectable in total tissue extracts. Alternatively, it is possible that some of the “constitutively bound” genes still move to different binding sites closer to the TSS, or are otherwise activated, either through combinatorial binding with other factors, or through chromatin accessibility regulation.

These results may have implications beyond the field of liver regeneration for understanding carcinogenesis during inflammatory conditions. Barash et al. proposed that chronic inflammation can increase hepatocyte genomic instability and that these genomically unstable hepatocytes can become tumorigenic following liver resection (Barash et al., 2010). The results of our study show the binding response of NF-κB to liver resection in the first 6 h post-resection. This NF-κB binding response can serve as a baseline from which to compare NF-κB response to resection during conditions of chronic inflammation caused by chronic liver diseases including alcohol use, obesity, hepatitis infection, or bile salt export pump deficiency (which causes inflammation and carcinogenesis in the absence of external, cancer-predisposing factors) (McGivern and Lemon, 2011; Sun and Karin, 2012; Iannelli et al., 2014; Kudo et al., 2014). Such a comparison could provide insights into how NF-κB contributes to hepatocellular carcinoma genesis during regeneration under chronic inflammatory conditions. Further study of these areas will be necessary to determine the dynamic contribution of NF-κB to such carcinogenesis.

Our approach has several strengths and weaknesses that are important to consider when interpreting our results. ChIP-chip can be considered as a targeted approach for promoter regions of genes; whereas ChIP-seq (next generation sequencing) gives a much more global view of transcription factor binding which includes the promoter regions and other regions within genes (Ho et al., 2011). ChIP-chip's higher coverage of the promoter regions of genes also gives a higher sensitivity than ChIP-seq unless a high read count is used. Additionally, both techniques share the limitations of low resolution and lack of cell-type specificity. Neither technique can differentiate with a base-pair specificity the true NF-κB binding sites within the bound regions. Recent techniques, such as ChIP-exo, may allow for identification of NF-κB bound motifs on the bound regions (Rhee and Pugh, 2012). The signal from whole-liver ChIP approaches likely comes predominantly from hepatocytes with lower abundance non-parenchymal cells likely contributing a lesser signal. Cell isolation techniques, such as those used in the ENCODE project, may be used in the future to identify the contributions of different cell types in the liver to NF-κB binding during regeneration (Winter et al., 2013). Additionally, our novel, dynamic pattern-based analysis allowed for unique insights into the dynamic switching mechanisms of NF-κB binding during the priming phase of liver regeneration.

This genome-wide pattern counts analysis revealed several dynamic NF-κB switches which occur post-PHx. Using this technique, we were able to identify dynamics of NF-κB binding within multiple pathways during liver regeneration. Additionally, we were able to identify a subset of genes that may be critical for healthy tissue function. This analysis strategy has wide application when analyzing transcription factor binding and can be used in other contexts as well as to understand dynamic transcriptional regulation.

## Author contributions

RV conceived and designed the experiments. BP performed the experiments. DC and LK analyzed the data. DC, JH, and RV wrote the manuscript.

### Conflict of interest statement

The authors declare that the research was conducted in the absence of any commercial or financial relationships that could be construed as a potential conflict of interest.

## Supplementary material

Figure S1 **Analysis of optimum FDR to maximize binding peaks identified and minimize false positives**. Maximum FDR cutoff was varied from 0 to 0.2 in increments of 0.01. An FDR cutoff at 0.05 allows for identification of a large number of NF-κB binding peaks while minimizing the expected number of false positives.

Figure S2 **Analysis of how FDR affects relative fraction of genes in each binding pattern**. Selecting an FDR cutoff between 0.01 and 0.20 does not dramatically change the relative fractions of genes in binding patterns 2-6. Increasing the FDR causes a decrease in the fraction of genes bound in pattern 1 and an increase in the fraction of genes bound in patter 7; however, at FDR cutoffs near 0.05, the fractions of genes in patterns 1 and 7 remain close to the 0.05 value.

Figure S3 **Differentially expressed genes (Fold change cutoff = 2) at 4 and 12 h post-PHx**.

Figure S4 **Scree plots of peak scores for NF-κB binding at 0, 1, and 6 h post-PHx**. At each time point, a cutoff value for peak score was chosen based on when a “knee” occurred in the scree plot and maintaining 500–1000 NF-κB peaks.

Figure S5 **NF-κB binding regions for olfactory receptor genes compared to DNAse1 hyperactivity sites from whole-liver tissue in mice from the ENCODE project**. Left panel: Accessibility of NF-κB binding location identified by comparison of NF-κB binding region to DNAse 1 hyperactivity sites for **(A)** Olr264, **(B)** Olr62, and **(C)** Olr92. Right Panel: Chromatin availability at the gene region of **(A)** Olr264, **(B)** Olr62, and **(C)** Olr92.

Figure S6 **Motif analysis of NF-κB binding sites**. The top binding motifs were matched to TRANSFAC to identify potential cofactors regulating gene expression with NF-κB at each time point (excluding those motifs involved in mainly DNA binding and polymerase activity).

Figure S7 **ChIP qPCR validation of NF-κB targets following PHx**.

Figure S8 **Quality control for NF-κB ChIP assay for measuring NF-κB binding post-PHx. (A)** Sonication optimization for the ChIP procedure. **(B)** The NF-κB p65 antibody used is specific for NF-κB binding. **(C)** Representative PCR gel showing ChIP of NF-κB on the SOD2 gene from 0-6 h post-PHx. **(D)** Representative PCR gel showing ChIP of NF-κB on the NOS2 gene from 0 to 6 h post-PHx. **(C)** Representative PCR gel showing ChIP of NF-κB on the G0S2 gene from 0 to 6 h post-PHx. **(C)** Representative PCR gel showing ChIP of NF-κB on the IGFBP1 gene from 0 to 6 h post-PHx. ChIP, NF-κB antibody-treated; IGG, negative control; Sonicated, positive control.
