# Supplementary figures and images for: A novel, dynamic pattern-based analysis of NF-κB binding during the priming phase of liver regeneration reveals switch-like functional regulation of target genes
Source: Front Physiol. 2015 Jul 7;6:189. doi: 10.3389/fphys.2015.00189 (PMC4493398; doi:10.3389/fphys.2015.00189)

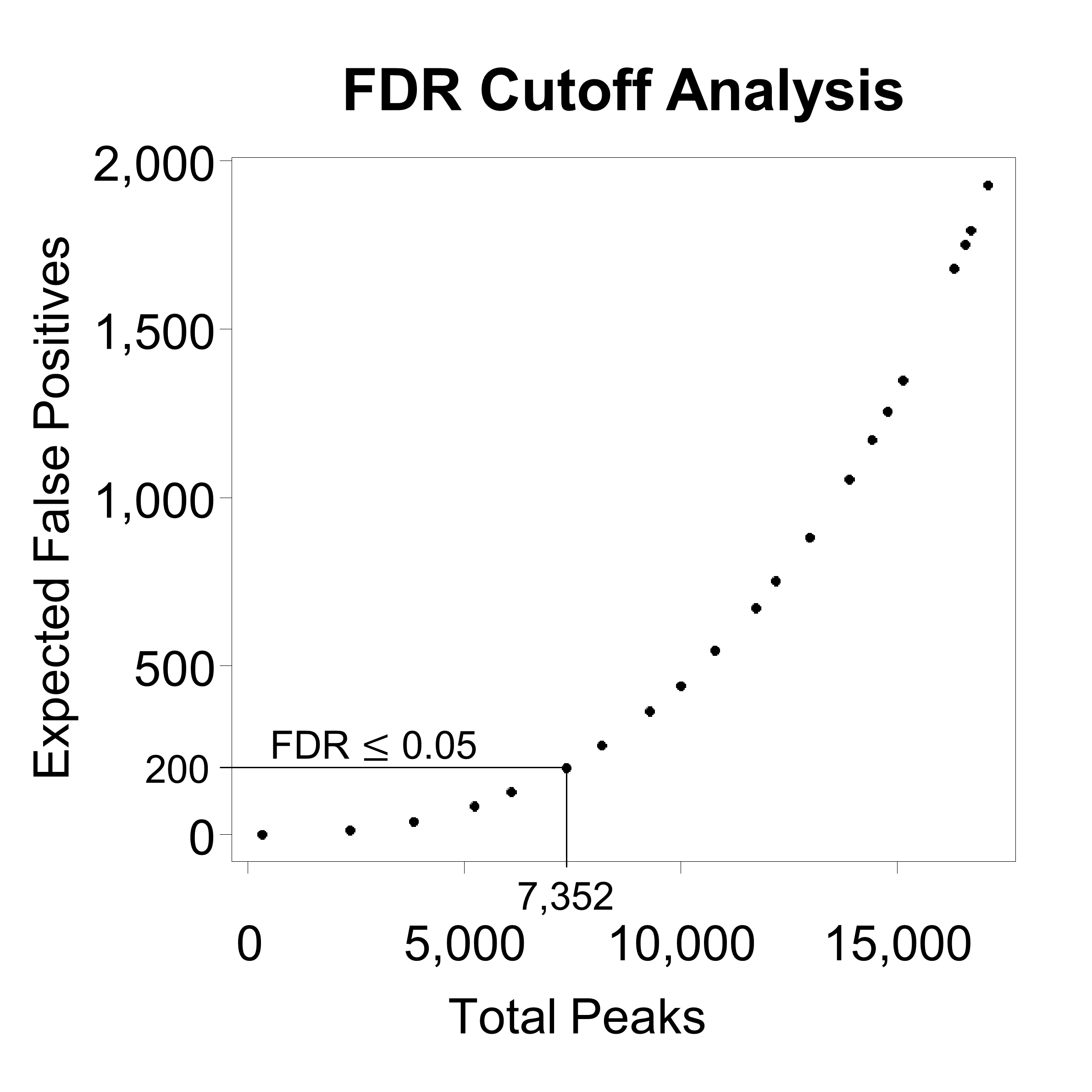

Supplement: Figure S1 — Analysis of optimum FDR to maximize binding peaks identified and minimize false positives. Maximum FDR cutoff was varied from 0 to 0.2 in increments of 0.01. An FDR cutoff at 0.05 allows for identification of a large number of NF-κB binding peaks while minimizing the expected number of false positives. [file Image1.TIF]

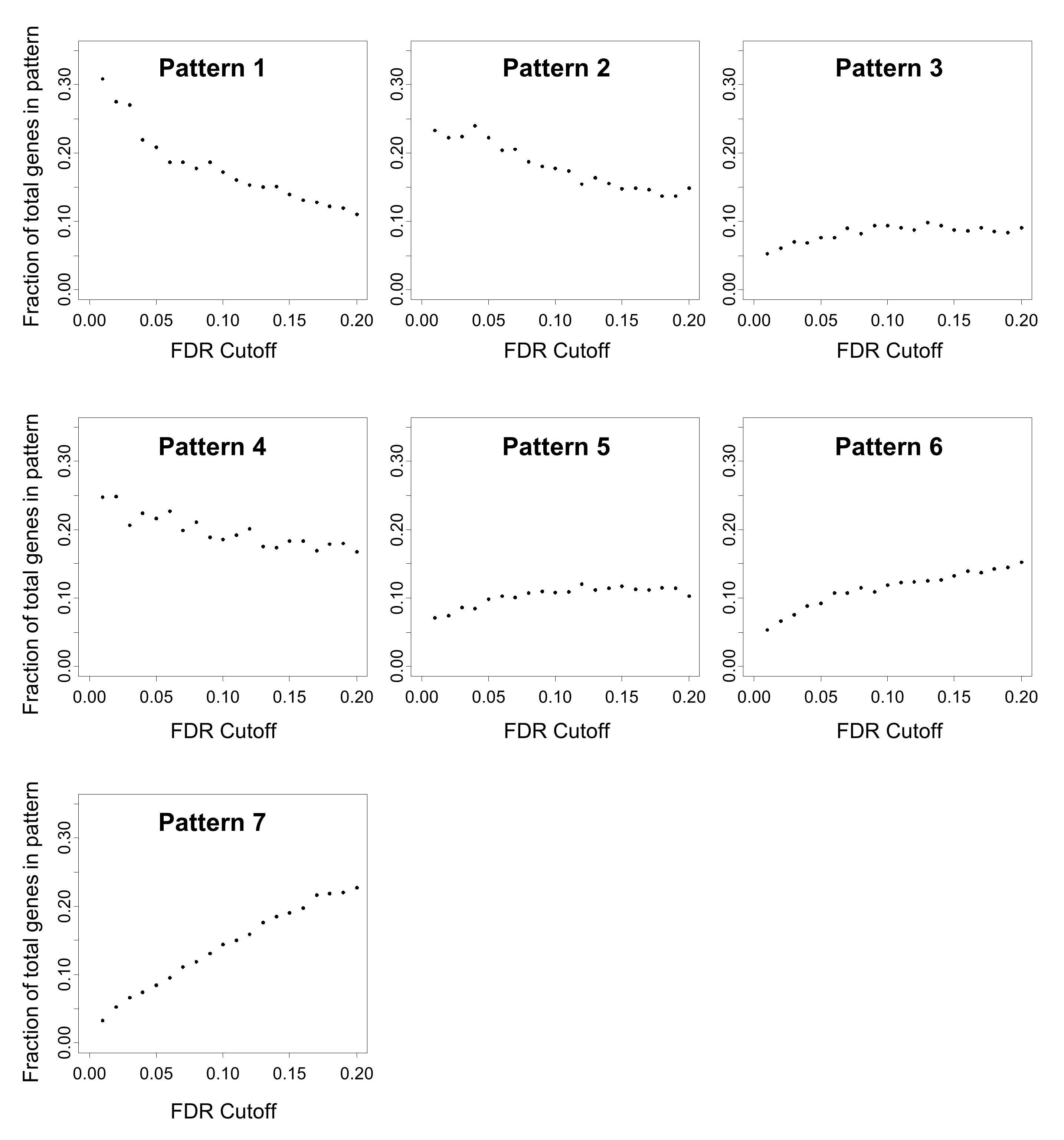

Supplement: Figure S2 — Analysis of how FDR affects relative fraction of genes in each binding pattern. Selecting an FDR cutoff between 0.01 and 0.20 does not dramatically change the relative fractions of genes in binding patterns 2-6. Increasing the FDR causes a decrease in the fraction of genes bound in pattern 1 and an increase in the fraction of genes bound in patter 7; however, at FDR cutoffs near 0.05, the fractions of genes in patterns 1 and 7 remain close to the 0.05 value. [file Image2.TIF]

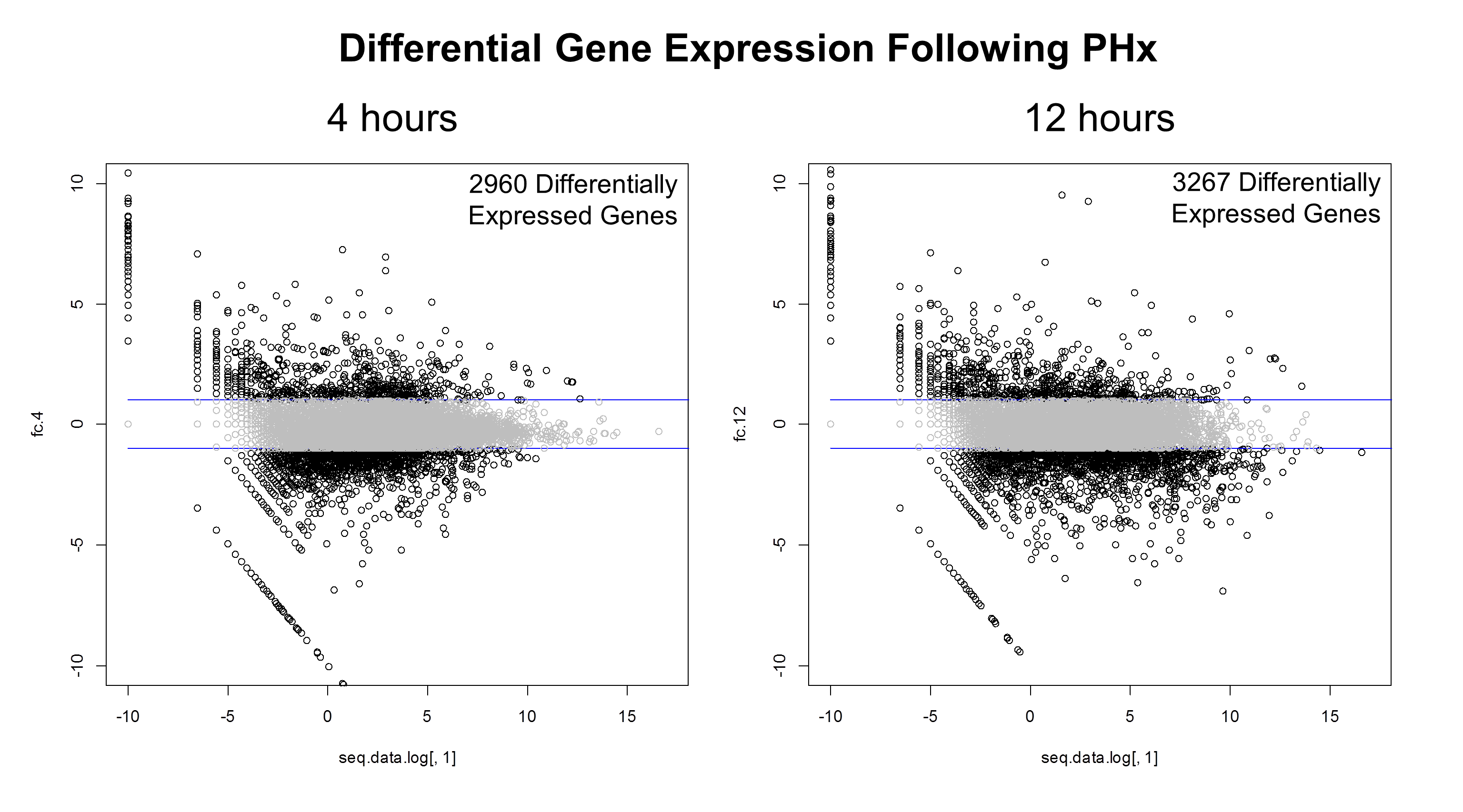

Supplement: Figure S3 — Differentially expressed genes (Fold change cutoff = 2) at 4 and 12 h post-PHx. [file Image3.TIF]

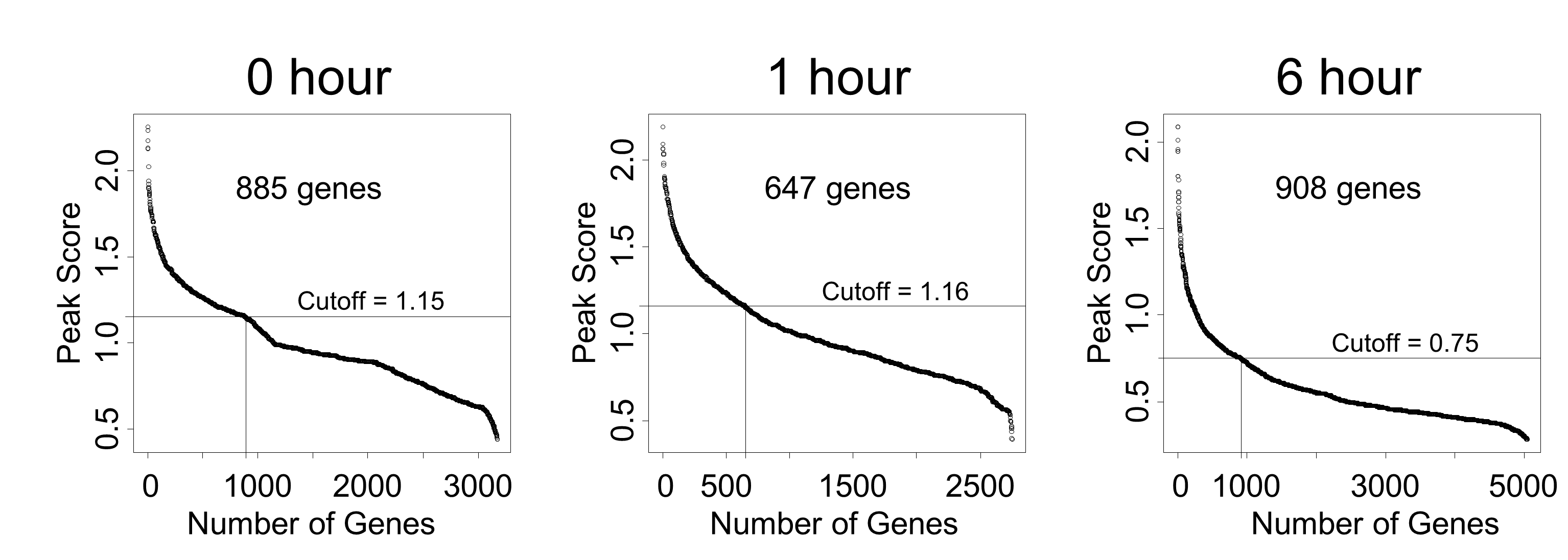

Supplement: Figure S4 — Scree plots of peak scores for NF-κB binding at 0, 1, and 6 h post-PHx. At each time point, a cutoff value for peak score was chosen based on when a “knee” occurred in the scree plot and maintaining 500–1000 NF-κB peaks. [file Image4.TIF]

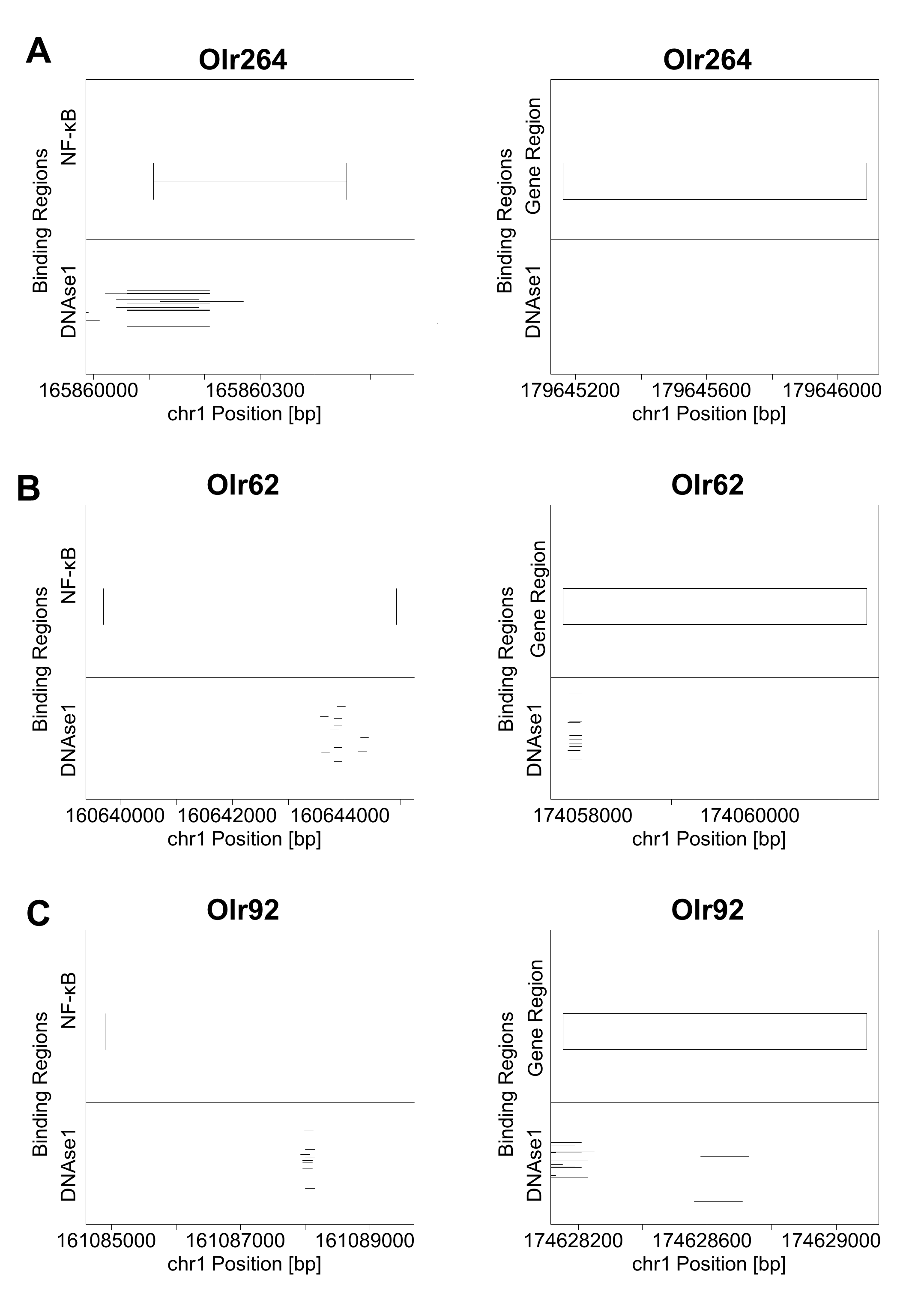

Supplement: Figure S5 — NF-κB binding regions for olfactory receptor genes compared to DNAse1 hyperactivity sites from whole-liver tissue in mice from the ENCODE project. Left panel: Accessibility of NF-κB binding location identified by comparison of NF-κB binding region to DNAse 1 hyperactivity sites for (A) Olr264, (B) Olr62, and (C) Olr92. Right Panel: Chromatin availability at the gene region of (A) Olr264, (B) Olr62, and (C) Olr92. [file Image5.TIF]

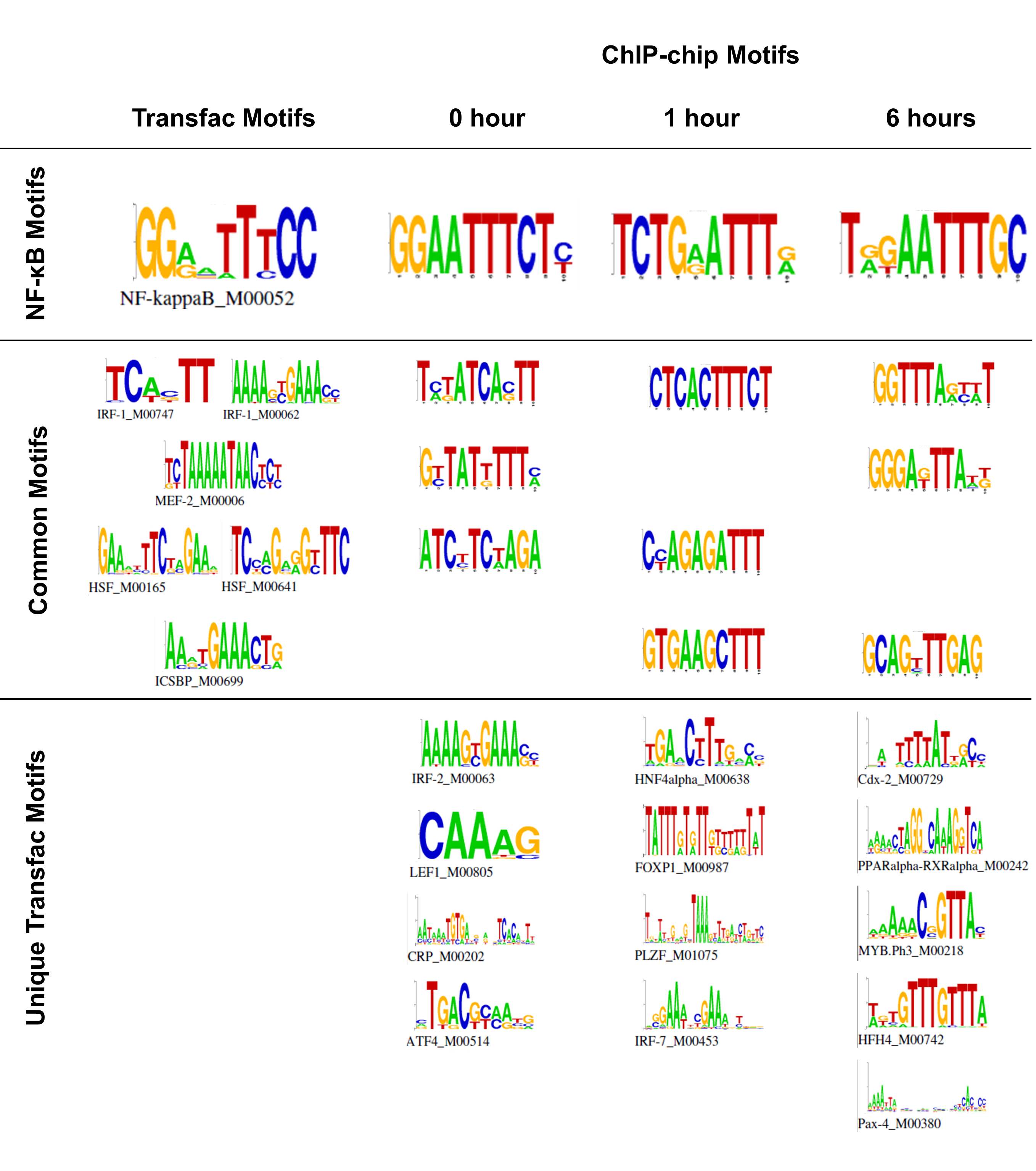

Supplement: Figure S6 — Motif analysis of NF-κB binding sites. The top binding motifs were matched to TRANSFAC to identify potential cofactors regulating gene expression with NF-κB at each time point (excluding those motifs involved in mainly DNA binding and polymerase activity). [file Image6.TIF]

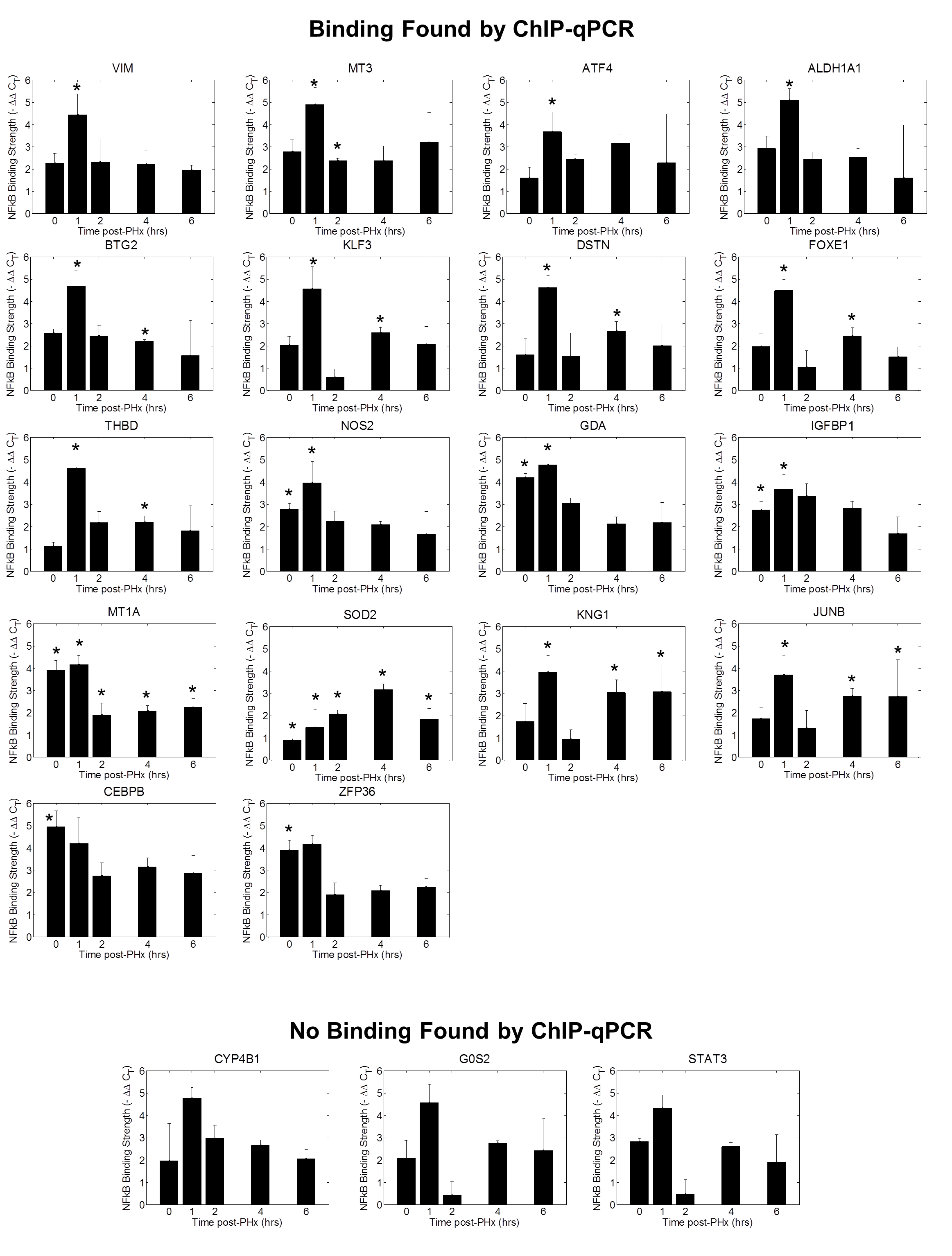

Supplement: Figure S7 — ChIP qPCR validation of NF-κB targets following PHx. [file Image7.TIF]

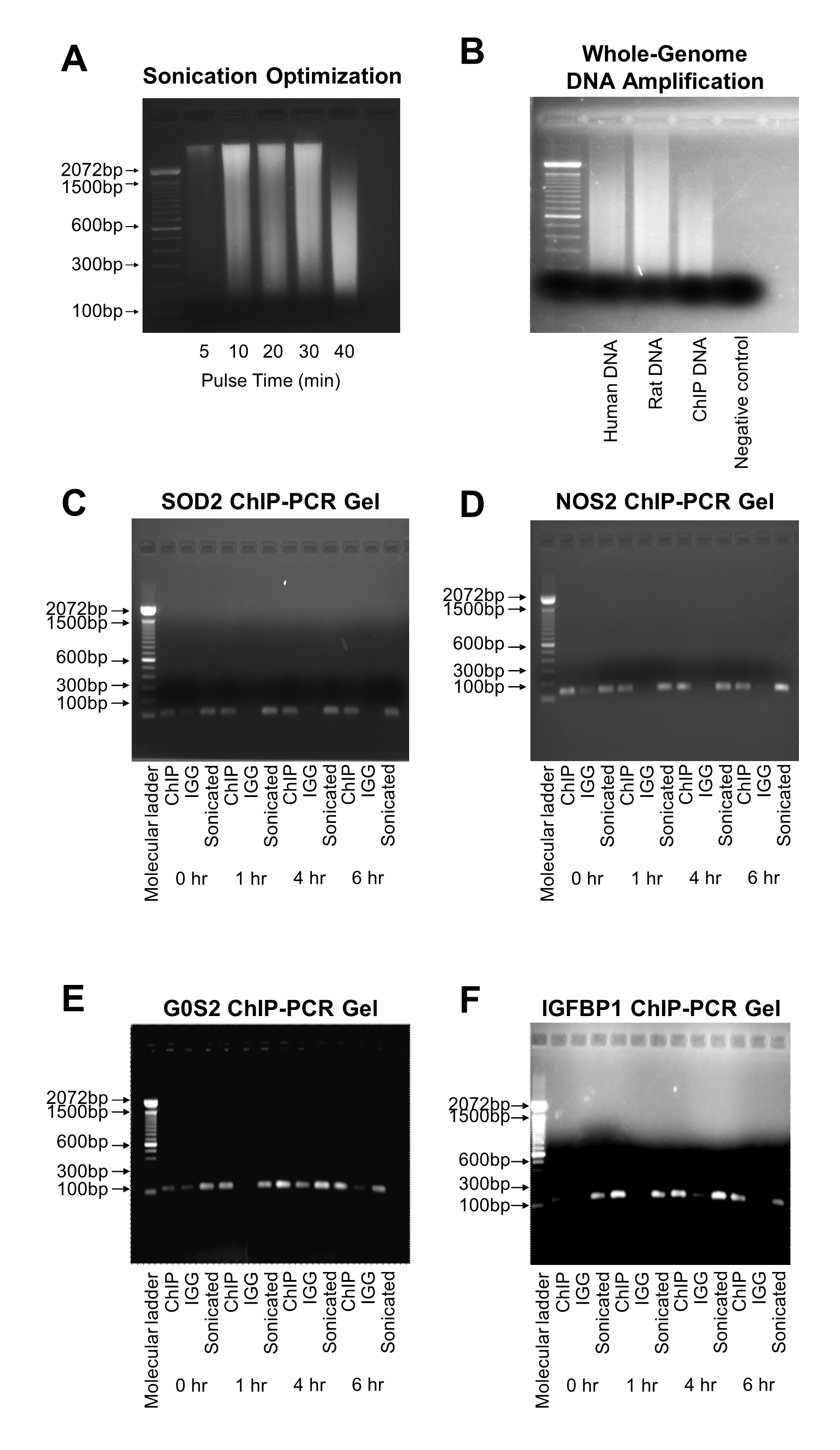

Supplement: Figure S8 — Quality control for NF-κB ChIP assay for measuring NF-κB binding post-PHx. (A) Sonication optimization for the ChIP procedure. (B) The NF-κB p65 antibody used is specific for NF-κB binding. (C) Representative PCR gel showing ChIP of NF-κB on the SOD2 gene from 0-6 h post-PHx. (D) Representative PCR gel showing ChIP of NF-κB on the NOS2 gene from 0 to 6 h post-PHx. (C) Representative PCR gel showing ChIP of NF-κB on the G0S2 gene from 0 to 6 h post-PHx. (C) Representative PCR gel showing ChIP of NF-κB on the IGFBP1 gene from 0 to 6 h post-PHx. ChIP, NF-κB antibody-treated; IGG, negative control; Sonicated, positive control. [file Image8.TIF]
