# Supplementary material for: A novel, dynamic pattern-based analysis of NF-κB binding during the priming phase of liver regeneration reveals switch-like functional regulation of target genes
Source: Front Physiol. 2015 Jul 7;6:189. doi: 10.3389/fphys.2015.00189 (PMC4493398; doi:10.3389/fphys.2015.00189)
Supplement: Supplementary file 9 [file DataSheet1.PDF]

Supplemental Table S1: FDR cutoff pathway analysis for Pattern 1

**FDR = 0.01**	**FDR = 0.02**	**FDR = 0.05**	**FDR = 0.10**
Acute inflammatory response	Acute inflammatory response	Acute inflammatory response	Acute Inflammatory Response
Argenine and proline metabolism			
Carboxylic acid boisynthesic process			
Lipoprotein metabolic process	Lipoprotein metabolic process		
Mitochondrion	Mitochondrion	Mitochondrion	Mitochondrial Part
Negative regulation of apoptosis		Negative regulation of apoptosis	
Nucleosome			
Plasma			
Regulation of blood vessel size		Regulation of blood vessel size	
Response to insulin stimulus	Response to insulin stimulus		
Ribosome	Ribosome	Ribosome	Ribosome
RNA Processing	RNA processing	RNA processing	
rRNA processing	rRNA processing	rRNA binding	
Triglyceride metabolic process			
Urea cycle	Urea cycle		
	Benzene and derivative metabolic process		
	Glucuronate metabolic processes		
	Peptidase inhibitor activity		
	Protein stabilization		
	Regulation of fibroblast proliferation		
	Ribosome assembly		
		Positive regulation of apoptosis	Induction of Apoptosis
		Positive regulation of protein kinase cascade	
		Response to xenobiotic stimulus	
			Androgen receptor binding
			Glucosamine metabolic process
			GPI anchor biosynthesis
			JmjC
			Negative regulation of protein binding

Supplemental Table S2: FDR cutoff pathway analysis for Pattern 5

FDR = 0.01	FDR = 0.02	FDR = 0.05	FDR = 0.10
Fibrinogen complex	Fibrinogen complex		
Gluconeogenesis			
Liver	Liver		
Positive regulation of adaptive immune response			
Protein catabolic process			
	Carboxylic acid binding	Carboxylic acid binding	Carboxylic acid binding
	Inflammatory response	Inflammatory response	
	Response to ethanol	Retinol metabolism	
		Olfactory receptor activity	Olfactory receptor activity
		Pheromone receptor activity	Pheromone receptor activity
			GPCR
			Monocarboxylic acid binding
			Phenylalanine metabolism
			Protein complex assembly

Supplemental Table S3: FDR cutoff pathway analysis for Pattern 7

FDR = 0.01	FDR = 0.02	FDR = 0.05	FDR = 0.10
Lung development			
	Acute inflammatory response	Acute inflammatory response	Acute inflammatory response
	Transcription factor complex		Transcription factor TFIIA complex
		Protein complex activity	Protein complex assembly
		Antimicrobial	Defense response to bacteria
		Liver	Liver
		Protein catabolic process	Protein catabolic process
		Regulation of JAK-STAT cascade	
			ATPase, AAA-type, core
			Negative regulation of apoptosis
			Negative regulation of DNA binding
			Sensory Perception of Smell
			UBL Conjugation

Supplemental Table S4: Primer Sequences

Gene Symbol	Foreword Sequence	Reverse Sequence
ALDH1A1	GTC TCG TGT GTG GGA CAT T	AAG GAC AGT GGC AAG GAG TG
ATF4	CTC TGC CTT GCT CTC CAA TTA	CCG GGG TAA TGA GCA GTA AA
BTG2	GAG CTC TGC TTG TGT CTG TCC	GCT CAG GGG AAA CAG AAC T
CEBPB	AGG AAA TCT TGG AGG GCT TC	CAG TGC TCC CTA TTC CCT CA
CYP4B1	TGT TAT TAC CTT TCC CGG TTT CCA AGC A	TTG GTC CTG CCA AGA CTG AAC CC
DSTN	TAG GGT GAC TCA TCT CAC TCA GCA	GCT GTG AAT GTC TAA ACT TGC ACT GAA GAG
FOXE1	ACT TCT TTG GAA GTC TGG AGG GCA	ACC TCG GTT TGC TTG GAG ACC TTT
G0S2	GGG CTT GTA AGA GTG CAT GAA GGT GAC A	TTT GCA GGC TAG GGT TGT GGA GTA CA
GDA	GCA CTC ATT AAT TGC CTG TAG TGC TTC	AGC AGA CCT GAA CAC CAG CTA ACA
IGFBP1	CGT CCT GTT GTG GTT TTG TG	CCT CCG CAC TAA GAA ACA TTG
JUNB	TAA GTC TTT GGC AGC TGT GTG GGA	TTG CTG GGT CTT CTT GGA AAC AGG CT
KLF3	GAG ATA GGG TCT TGA TAT GTA GCC CAG G	TTC TCC TGA GAA AGT GGC CC
KNG1	TGC TTT GAC CCT TAG TAA CCC GGA	TGT AGA CTC ACT CCC AGG ACA GTC A
MT1A	AGG CAT CCA GGT TGA GTC TG	GGC ATT AAC GGC TGG TTT TA
MT3	GCC GGG CTC CTA GTA CTT TT	AGG CTC AAC AAG CAG AAA CAG
NOS2	GGA ACC ATG GGA TGA TGA GT	TAC ATG GCA TGG GAT TTT CC
SOD2	CCG GAA GAG GAC ACA GCT GAG ATC ATT GTA	TGA GGA AAG GTG GCT CTG ACG GTAT T
STAT3	ACC AAG ATA GAA CTC ACT GAT GGG C	TGA ACC CAG ATC TCT GGC ACT CAT GT
THBD	GCC TGT AGG TAA GCC CAT GA	CAG GAC CAC CAG CCT AAG AG
VIM	TTC TTT CTC AGC ACC CAA GG	GGA TCG AGC ACA GTC CTG TTA
ZPF36	TCA CGG GAC CAG CCC AGG AA	TGT GTG TAT GTG TGT GTG TGT GTG CG
